# Climate change from the perspective of the New Public Health

**DOI:** 10.3389/fpubh.2025.1620117

**Published:** 2025-07-17

**Authors:** Toni Buterin, Iva Rinčić, Amir Muzur, Robert Doričić

**Affiliations:** ^1^Faculty of Health Studies, University of Rijeka, Rijeka, Croatia; ^2^Faculty of Medicine, University of Rijeka, Rijeka, Croatia

**Keywords:** climate change, the New Public Health, ecological crisis, society, adaptation

## Abstract

The modern-day ecological crisis and gradual degradation of the environment, mostly due to anthropogenic effects, surpass other contemporary societal issues. Despite being largely perceived through a (bio)medical lens, the complexity of climate change as a topic is seen in different trends concerning its impact on the living world. These include historical, economic, cultural and social dimensions. Therefore, there is a need for an integrated and interdisciplinary approach resulting in more comprehensive measures to allow society to recover, but which also exploit the positive potential of climate change, through models and methods that the New Public Health can provide. Starting from the definition of the New Public Health, this paper combines and connects two topics, New Public Health and climate change, that are rarely explored together in the literature. The aim is to fill the gaps in the public health literature, where climate change is frequently viewed solely as a medical or health issue; here, we frame it as a critical challenge encompassing social, humanistic, and environmental dimensions. In addition, we offer a conceptual contribution that emphasizes their interconnection within the context of contemporary challenges.

## Introduction

The World Health Organisation emphasises that climate change represents one of the greatest global threats of our time (1, 2). The human impact on the natural environment has resulted in (ir)reversible processes, most often associated with industry (3, 4). Preventive measures and strategic planning therefore have a positive effect on the population’s health by reducing the anthropogenic impact of environmental pollution (5). Although the greatest effect of climate change is on human health, it should not be viewed exclusively in medical terms. By broadening perspectives, moving away from an anthropocentric approach and integrating social, human, technical and natural sciences, we obtain a bio-social view of this public health and environmental issue. In this context, results can be more complete, and concrete action can be taken in line with climate policy (6, 7) in an interdisciplinary environment on which the foundations of the New Public Health concept rest (8, 9).

Environmental protection and climate change prevention policies are largely based on strategies, acts and measures to reduce pollution from industry, which has left a significant mark on the environment, population health, and even mortality (10, 11). We cannot deny the thesis that the widespread use of fossil fuels is a major contributor to climate change. Fossil fuels are the primary factor behind climate change that currently harm human health, wellbeing, and ecosystems. This point must be taken into account because without a full understanding of the causes and consequences of climate change, we cannot develop more effective solutions. While there are proponents of fossil fuel use, some sources emphasize their benefits, particularly in driving economic progress and technological advancement, it is crucial to recognize that these views are widely debated within the scientific community. Many researchers advocate for a transition to renewable energy to mitigate environmental and health risks (12, 13), and this perspective has far more significant implications for the field of the New Public Health.

In this regard, an important contribution to the adaptation of society and the environment to climate change is provided by the synergy and integration of different disciplines. As industrial pollution and anthropogenic impacts are the main causes of global warming and climate change, the consequences for humans are mainly related to the biomedical aspects of their impact on health, which are mostly on the respiratory and cardiovascular systems. These impacts are also associated with the development of neoplasms, skin diseases, and neurodegenerative and endocrine disorders (14–16). The main subject of research in this area is air pollution, because it affects the largest number of systems in the human body (17). However, the impact on water and soil is neglected, though numerous ecological studies on water and soil pollution show significant consequences for public health (18). As a result, solutions are offered in terms of technical forms of reducing pollution (19, 20). Studies to date indicate that most research linking climate change and public health is focused on the deterioration of human health (21). Epidemiological data arising from human activities that cause climate change provide strong evidence of their impact on populations. The fact remains that Epidemiology needs to keep evolving by creating innovative methods and incorporating insights from other fields like geography and climatology (22). Numerous studies report increases in mortality and morbidity associated with air pollution (23), and such data combine descriptive and analytical approaches what play a crucial role in identifying causal links between environmental changes and health outcomes. Emphasizing these epidemiological insights is essential for guiding public health policies and interventions that address the real health threats posed by climate change, further reinforcing the need for an integrated and prevention oriented approach as advocated by the New Public Health.

Some research, however, suggests that more decisive responses are needed to address the challenges of climate change, which is adding further strain to the existing weaknesses in health systems. This not only affects health but is prompting a reassessment of priorities. The most effective responses appear to be strengthening key factors such as environmental management, and systems for monitoring and controlling the consequences of natural disasters and changes in infectious disease patterns. A more proactive approach to decision-making is called for. These interventions are often not new but rather build on existing tools that are underutilized due to key barriers such as a lack of political will or financial resources. Climate change therefore requires an intensification of preventive measures in public health, and this task should take a central place in sustainable development strategies (24).

The philosophy of the New Public Health seeks to balance the excessive emphasis on health preservation priorities exclusively through the biomedical aspect (8). Such an approach integrates various disciplines that are interrelated at the human-society level. Based on the application of a wide range of scientific and technological methods and evidence-based management models of various professional, political and ethical dimensions, it is possible to improve attitudes toward prevention and protection of individual and societal health in a broader form (8).

## Methods

Using a combined approach of a literature review and the description method, the key characteristics of the publications published so far on the topic of the New Public Health and climate change have been structured, synthesized and described in detail. Given that only a small amount of literature that directly connects these two topics has been found, the research question arises of whether there is a need for newer public health approaches and paradigms, and what are the key elements, challenges and interventions needed which are directly or indirectly related to climate change. With this critical review, we aim to identify the vulnerable dimensions of society, and trends and limitations in the integration of problems caused by climate change in the philosophy of the New Public Health by using more explicit present-day examples.

## Results

The trend in the contemporary development of the New Public Health concept is interesting to follow. In the late 20^th^ century, public health faced numerous challenges but also made significant progress. Each crisis in public health and weakening of it demonstrated the need for new approaches to dealing with a particular problem. The increase in chronic diseases and the increased manifestation of neoplasms required new preventive strategies, while infectious diseases remained the primary global threat. Therefore, scientists called for the reintegration of public health into clinical practice and advocated the need to consider medical history and better education (25, 26).

At the turn of the 20^th^ and 21^st^ centuries, globalization, urbanization and environmental issues further highlighted the need for integrated approaches to public health focused on prevention, education and strengthening health systems worldwide. Effective public health required building on the changes in society over the previous decade in moving toward a new approach to public health. It was necessary to move away from the dominance of the medical model and turn toward a socio-environmental model that would be more comprehensive in terms of sustainable health improvement (27).

In the 21^st^ century, public health has faced numerous challenges that continue to threaten population health and that have been marginalized until now. The need has arisen to create priorities, especially improving basic healthcare for vulnerable groups, expanding cooperation among health partners, establishing broader disease surveillance, instituting early warning and rapid response systems, reducing morbidity and mortality (for mothers, newborns and children under five years of age), but also providing psychosocial support to victims (28). Environmental degradation, wars and terrorism, growing economic inequality, and weakening social ties have been the main threats. Although public health was once primarily focused on medical approaches, there is a growing need for multidisciplinary approaches, with an emphasis on the social, economic and environmental factors that shape people’s health (29). It is necessary to go beyond the traditional approach to health by including social factors that can affect the public health picture of the population (30). At the time, milestones in the development of public health have been presented through historical legacy, current status and innovative approaches to health promotion, but with an emphasis on striving to create newer approaches to solving problems at some future time that will again create new challenges (31).

The direct impact of climate change on population health, but also on society, is visible in meteorotropic diseases, while the indirect effect of weather variations can be seen in their impact on food production, the availability of drinking water, infrastructure, mental health and the transmission of infectious diseases. Many of the consequences of a polluted environment which can have an impact on health are visible only after some time or appear as longer-term consequences of chronic exposure. Other health risks include increasingly frequent climate variations that cause high-frequency and intense heatwaves, temperature oscillations, extreme precipitation changes and even fires (32). Just as climatic conditions can affect the causative agents of infectious diseases, they can also affect their carriers and vectors. The occurrence of vectors and the spread of infection are interrelated with climate change, which leads to the expansion of their habitats, but also the transmission routes of vector-borne diseases (33, 34). We are increasingly witnessing exceptional cases of flooding of coastlines, including island and coastal towns, while flora and fauna are changing their habitats depending on the need to adapt to new climate conditions. The complexity of the topic of climate change requires an interdisciplinary approach. Identifying vulnerable dimensions of society besides health, such as the economy or culture, can show different trends in the impact of climate change on society. Starting from the local community, it is possible to act at a global level – with models and methods for mitigating climate change and adapting and coexisting with it when it is already present – by expanding and integrating different scientific disciplines for more comprehensive action (35).

For these reasons, there is an increasing call for a redefinition of global public health. The challenges of the 21^st^ century have resulted in threats that may be caused by climate change. These require, among other things, political action and systemic changes (36). We are witnessing how global climate summits and conferences are increasingly taking the form of public spectacles characterized by elitist approaches to organization, often lacking in concrete outcomes or meaningful agreements. Of course, exceptions exist, and certain individuals and initiatives do stand out even within such constrained settings. It is important not to overlook the pioneering efforts of the 1972 United Nations Conference on the Human Environment in Stockholm, which addressed environmental protection, including early concerns about climate change (37). Equally significant are the ongoing efforts of the *Intergovernmental Panel on Climate Change* (IPCC), which plays a central and irreplaceable role in understanding and managing climate-related challenges through a multidisciplinary approach at the global level (38).

We can certainly resort to such multidisciplinary solutions through the lens of the New Public Health, which has in recent times been keeping pace with global challenges. The basic prerequisites have already been created and are being developed from year to year, having significant importance in dealing with climate change (9).

Therefore, it seems crucial to: (I) increase the availability of information on climate change from different scientific and professional perspectives; (II) identify vulnerable bio-social elements of society in relation to climate change; (III) increase the awareness and ability of the population to recover from the negative effects of climate change by popularising science; (IV) strengthen the social system by adapting to climate change and exploiting its possible positive potentials.

A comprehensive definition of health that refers to a state of complete physical, mental and social well-being, and not merely the absence of disease or infirmity (39), underlines the need to consider the broader picture of the consequences of climate change rather than just focusing on its medical ones. Climate change is a complex issue that shows different trends of influence across the historical, economic, cultural, economic, legal, social and political dimensions of society. Depending on the values of an individual or group, the focus of combating climate change, adapting to it or promoting climate action will depend on perceptions and needs (6). When seen from a cultural approach, where culture and tradition intertwine through a dynamic process of adaptation and transformation, climate change affects the culture, identity and material heritage of communities, while cultural patterns shape the perception of the population and thus (their) responses to environmental challenges (40). Climate change can also be viewed from the sociological perspective – for example, through rapidly increasing climate-driven migrations that (un)consciously change demographic structures, causing social tensions and impacting the identity of communities (and which may be interrelated with the previously mentioned cultural perspective). At the same time, social responses to migration result in new needs for adaptation and solidarity toward vulnerable populations (41, 42). Economic activity is not devoid of consequences in terms of the impact of climate change either. This is particularly true for the tourism sector, where both positive and negative consequences can be seen. On the one hand, a warmer climate can prolong the tourist season in certain regions and attract tourists, which can boost economic growth. On the other hand, extreme weather conditions, rising sea levels and the destruction of natural resources can lead to the migration of local populations (which can again be linked to the previously mentioned sociological perspective) and the loss of the attractiveness of tourist destinations, but also cause a loss of tourist revenues (43–45). Agronomy and agriculture should not be ignored either. In this area, there is a possibility of improving the conditions for growing certain crops in new regions where this has not previously been possible. However, on the other hand, there is a danger of droughts, floods and fires that can reduce yields, increase production costs and threaten food security (46, 47). The health component largely encompasses the impact on human organ systems and the spread of diseases. However, it is equally important to pay attention to the social dimension of health – especially regarding working environment and conditions (48, 49). There are other fields and disciplines such as bioethics, ecoethics and environmental ethics that contribute to understanding the problem and that attempt to participate in the study of climate change through an interdisciplinary approach that comprises ethics, values and responsibility both in real time and for the future (50–52).

In the case of climate change, it is clear that resources, which have already come under significant strain, are being additionally burdened. This further highlights the importance of integrated surveillance systems for future adaptations (21). Therefore, the intertwining and interdependence of the previously identified vulnerable dimensions of society affected by climate change require the constant updating and modernisation of a discipline like public health – regardless of whether we call it *Public Health*, *New Public Health* or *Newer Public Health*. Interventions and strategies for adaptation to and/or mitigation of the impact of climate change will need to adjust to new challenges that are already present, but also to those that are coming. It is of great importance to adopt innovative forms, increase awareness and the ability of society to recover, and to exploit any positive potentials through appropriate communication and more effective actions that keep pace with the times (53, 54). These can be found in the guiding principles of the New Public Health philosophy (9).

## Conclusion

The challenges posed by climate change are most often associated with health issues and are rarely considered in the specific context of the New Public Health. Although there are clear and scientifically based links between climate change and the philosophy of the New Public Health, we believe that scientific discourse needs to emphasize them more explicitly and more frequently. This Mini Review explores the relationship between climate change and the New Public Health, two distinct yet interconnected phenomena that are still rarely and insufficiently addressed together in current literature. In light of the escalating global health threat posed by climate change, the review underscores the relevance of the New Public Health paradigm, with particular emphasis on interdisciplinarity, community engagement, and proactive protection strategies. This approach offers a valuable framework for both adaptation and mitigation efforts. Key priorities include: improving the accessibility of climate change-related information across scientific and professional domains; identifying vulnerable bio-social segments of society to better inform protective interventions; promoting scientific literacy and public engagement to enhance societal resilience; and strengthening the social system through adaptive strategies that also acknowledge and harness the potential positive aspects of climate change.

Climate change brings risks that go beyond the medical aspect and encompass other ecological, economic, business and social issues. The future development of public health should include more systematic research and integration of the impacts of climate change into the broader spheres of protection and adaptation policies and strategies. We need to encourage an inter/multi-disciplinary approach that will connect experts from different fields in order to provide comprehensive solutions to the challenges posed by the climate crisis. This paper encourages further academic discussion about the need for frequent transformation and modernization of public health in the era of climate change, and provides a basis for the development of policies aimed at longer-term sustainability. Its importance lies in its contribution to the understanding of the interdependence of the environment and people and the need to integrate all the factors that create their interrelationship within the discipline of the New Public Health.
